# Age-Related Changes in Inter-Network Connectivity by Component Analysis

**DOI:** 10.3389/fnagi.2015.00237

**Published:** 2015-12-24

**Authors:** Christian La, Pouria Mossahebi, Veena A. Nair, Barbara B. Bendlin, Rasmus Birn, Mary E. Meyerand, Vivek Prabhakaran

**Affiliations:** ^1^Neuroscience Training Program, University of Wisconsin-MadisonMadison, WI, USA; ^2^Department of Radiology, University of Wisconsin-MadisonMadison, WI, USA; ^3^Department of Medicine, University of Wisconsin-MadisonMadison, WI, USA; ^4^Department of Psychiatry, University of Wisconsin-MadisonMadison, WI, USA; ^5^Department of Medical Physics, University of Wisconsin-MadisonMadison, WI, USA; ^6^Department of Bio-Medical Engineering, University of Wisconsin-MadisonMadison, WI, USA

**Keywords:** functional connectivity, default-mode network, fMRI, ICA, aging, dedifferentiation

## Abstract

Healthy aging is associated with brain changes that reflect an alteration to a functional unit in response to the available resources and architecture. Even before the onset of noticeable cognitive decline, the neural scaffolds underlying cognitive function undergo considerable change. Prior studies have suggested a disruption of the connectivity pattern within the “default-mode” network (DMN), and more specifically a disruption of the anterio-posterior connectivity. In this study, we explored the effects of aging on within-network connectivity of three DMN subnetworks: a posterior DMN (pDMN), an anterior DMN (aDMN), and a ventral DMN (vDMN); as well as between-network connectivity during resting-state. Using groupICA on 43 young and 43 older healthy adults, we showed a reduction of network co-activation in two of the DMN subnetworks (pDMN and aDMN) and demonstrated a difference in between-component connectivity levels. The older group exhibited more numerous high-correlation pairs (*Pearson's rho* > 0.3, Number of comp-pairs = 46) in comparison to the young group (Number of comp-pairs = 34), suggesting a more connected/less segregated cortical system. Moreover, three component-pairs exhibited statistically significant differences between the two populations. Visual areas V2–V1 and V2–V4 were more correlated in the older adults, while aDMN–pDMN correlation decreased with aging. The increase in the number of high-correlation component-pairs and the elevated correlation in the visual areas are consistent with the prior hypothesis that aging is associated with a reduction of functional segregation. However, the aDMN-pDMN dis-connectivity may be occurring under a different mechanism, a mechanism more related to a breakdown of structural integrity along the anterio-posterior axis.

## Introduction

As our brain grows to maturity from childhood through adolescence and adulthood, it evolves to adapt to the ever changing external task demand and to its internal environment. Advanced aging is also often associated with cognitive decline. Speed of processing (Andrews-Hanna et al., 2007), executive functions (Damoiseaux et al., 2008) and memory function (He et al., 2012; Vidal-Piñeiro et al., 2014) become compromised. However, even before the appearance of noticeable decline in those abilities, the neural architecture underlying these processes has likely already undergone considerable change (Paulsen et al., 2004; Hampel et al., 2008; Callaghan et al., 2014). The “compensation related utilization of neural circuits hypothesis” (CRUNCH) posits that older adults may engage control at lower levels of task load to preserve performance, making age-related differences difficult to detect in behavioral measures where task load is lower than one's cognitive limit despite large differences in underlying processing (Reuter-Lorenz and Lustig, 2005; Reuter-Lorenz and Cappell, 2008). Even when older adults do not show behavioral impairments, neural measures often indicate impaired or at least differential processing.

Several neuroimaging studies have shown age-related cortical network re-structuring in aging, or more specifically, a reduction of the specialization of the hemispheres with recruitment of the contralateral homolog for a more bilateral activation (Reuter-Lorenz et al., 2000; Cabeza, 2002). Furthermore, the “scaffolding theory of aging and cognition” (STAC; Park and Reuter-Lorenz, 2009) extends on the CRUNCH hypothesis (Reuter-Lorenz and Lustig, 2005; Reuter-Lorenz and Cappell, 2008) and describes a recruitment of proximal and/or distal brain structures as a consequence of decline of the functional neuronal unit. This forges an alternative neural circuit in order to preserve function. These hypotheses are further supported by studies which show a reduction of functional specialization, or “dedifferentiation” in aging (Baltes and Lindenberger, 1997; Li et al., 2001; Park et al., 2004), where the functional unit exhibits reduced functional specificity in order to be recruited in the goal-directed behavior. The phenomenon has been observed in multiple systems including the ventral visual system (Park et al., 2004, 2012), motor system (Carp et al., 2011a; Bernard and Seidler, 2012) and other higher-order systems (Carp et al., 2011b).

It has been further hypothesized that aside from local changes, aging involves alterations in the integration of regional brain activity (functional brain connectivity). For the investigation of these underlying changes, resting-state fMRI (rs-fMRI) analysis has proven particularly advantageous. The condition of *rest* is defined as a state where no active participation in a task is required. Nevertheless, functional connectivity and network integrity can be reliably assessed in the resting state (Birn et al., 2013; Patriat et al., 2013). In addition, it has been suggested that these resting-state networks (RSNs) may reflect an intrinsic property of brain functional organization that serves to stabilize brain ensembles (Buckner and Vincent, 2007; Raichle and Snyder, 2007).

Functional connectivity (Friston et al., 1993) is an approach used in the analysis of rs-fMRI. It measures the correlation, or level of synchronization between signals from distal brain regions (Biswal et al., 1995), with high synchronization suggesting shared functionality. This approach allows for the depiction and identification of spatially distinct but functionally related regions, forming diverse, but robust intrinsic functional networks of the cortical system (i.e., RSNs; Beckmann et al., 2005; Damoiseaux et al., 2006; Fox and Raichle, 2007). The “default-mode” network (DMN) is a well-investigated RSN involving a set of regions previously observed to consistently deactivate during an external task and become active in the resting condition (Shulman et al., 1997; Binder et al., 1999; Gusnard et al., 2001a; Raichle et al., 2001; Fox and Raichle, 2007). Comprising the precuneus/posterior cingulate cortex (pC/PCC) complex, the medial prefrontal cortex (mPFC), and bilateral inferior parietal lobules (IPLs) at its core, this network has been suggested to serve role in mind-wandering (Raichle et al., 2001; Mason et al., 2007), introspection, memory, and self-referential processes (Ochsner et al., 2004; Schmitz et al., 2004; Buckner et al., 2008; Qin and Northoff, 2011). These functions may be served by two primary subsystems of the DMN (Buckner et al., 2008): a posterior subsystem including the pC/PCC, bilateral IPL and the MTL for episodic memory retrieval (Wagner et al., 2005), and an anterior subsystem with the anterior regions of the DMN (the dorsal and ventral mPFC), and including the pC/PCC complex, involved in self-referential mental thoughts (Gusnard et al., 2001b; Schmitz et al., 2004; Johnson et al., 2005).

From a cognitive neuroscience point of view, the DMN has received particular attention because of its lack of activation during attention-demanding tasks (Shulman et al., 1997; Mazoyer et al., 2001). However, among the elderly subjects, the DMN exhibited less deactivation during semantic classification (Lustig et al., 2003), visual oddball (Persson et al., 2007) and memory (Grady et al., 2006) tasks among others. Functional connectivity measures within the DMN also have shown age-associated reductions (Damoiseaux et al., 2008; Koch et al., 2010; Tomasi and Volkow, 2012). Additionally, this network has become a primary focus of study as biological markers for abnormalities in the brain for a wide spectrum of psychiatric disorders. (see Buckner et al., 2008; Greicius, 2008; Broyd et al., 2009for reviews). Furthermore, an investigation of connectivity using seed-based pair-wise correlation showed a large-scale disruption within this brain system in advanced aging, with a reduction of the anterio-posterior connectivity between the nodes of the medial prefrontal cortex and the posterior cingulate cortex (Andrews-Hanna et al., 2007).

In principle, time-course correlation with seed-based analysis should provide high sensitivity to true differences in the correlations between specific regions. However, this method is limited by a potential variation in the localization of these regions across subjects and the influence of structural spatial confound among other drawbacks that include high susceptibility to motion (Cole et al., 2010; Van Dijk et al., 2012). On the other hand, independent component analysis (ICA), a data-driven method, allows for a decomposition of multivariate signal into independent non-Gaussian signals, free of pre-determined assumptions, possibly confounding results and interpretations, providing a more comprehensive assessment of correlation variations. In addition, RSNs identified by ICA can be less prone to artefactual effects from noise (Birn et al., 2008; Murphy et al., 2009; Cole et al., 2010), due to its ability to separate and isolate non-RSN components.

In this study, we revisited the disruption of DMN functional connectivity in aging, specifically the reduction of the anterior to posterior functional connectivity, but with a more comprehensive, data-driven approach of an analysis of the components identified using ICA. We hypothesized that the previously suggested reduction of network co-activation within the posterior and anterior component of the DMN, describing a reduction of DMN functional integrity in aging, can be replicated here. But, we further postulated that through the model-free component analysis approach, the age-related reduction of anterio-posterior connectivity between the anterior and posterior regions within the DMN can be demonstrated, suggesting a significant and consistent disconnection between the two subsystems persistent with advanced age.

## Materials and methods

### Participants

A total of eighty-six participants were recruited into the study and separated into two groups: young adults (18–30 years old) and older adults (>50 years old). Details of participant characteristics are presented in Table 1. Each participant was invited to complete a MR scanning session including a 10-min resting-state scan and a high resolution structural scan (parameters described in the next section). Participants in this study expressed no history or signs of neurological or psychiatric disorders, and provided full written informed consent in compliance with the UW-Madison Health Sciences Institutional Review Board (IRB).

**Table 1:** **Subject demographics table**.

**Population group**	**Sample size (n)**	**Age in years (mean± stddev)**	**Gender M: F**	**Handedness L: R: A**	**Education (in years)**
Young adults (< 30 y.o.)	43	22.5 ± 2.7	23: 20	4: 39: 0	17.2 ± 2.2
Older adults (>50 y.o.)	43	60.9 ± 8.1	22: 21	4: 37: 2	16.8 ± 2.7

### MRI acquisition

Neuroimaging data were collected at the University of Wisconsin-Madison, using a 3.0-Tesla GE Discovery MR750 scanner (GE Healthcare, Waukesha, WI), equipped with an 8-channel head coil. A 10-min resting state fMRI scan was acquired while the subject was instructed to lie still and relax with their eyes closed for the duration of the scan with the following parameters: single-shot T2^*^-weighted gradient-echo echo planar imaging, with 40 sagittal slices, TR = 2.6 s, TE = 22 ms, FOV = 224 mm, flip angle = 60°, isotropic 3.5 mm^3^ voxel. Subjects were reminded to hold still and minimize head motion. The 3D high-resolution axial structural scan was acquired using a T1-weighted IR-prepared FSPGR BRAVO sequence with 156 slices, isotropic 1 mm^3^, over a 256 × 256 matrix, TR = 8.132 ms, TE = 3.18 ms, TI = 450 ms, FOV = 256 mm, flip angle = 12°. Earplugs and foam padding were used to attenuate scanner noise and minimize head movement, respectively.

### Data pre-processing and isolation of independent components

Pre-processing of imaging data was performed using SPM8 (Wellcome Trust Centre for Neuroimaging, University of College London, UK), which included removal of the first 10 time-points to allow magnetization to reach steady state, slice-timing correction, and motion-correction. Dataset with motion exceeding 2-mm in any of the three cardinal directions (i.e., x, y, z) were removed from analysis. Data spikes were removed using AFNI's 3dDespike (http://afni.nimh.nih.gov/afni/). The functional images were then normalized to the MNI-152 template using non-linear transformation in SPM8, and smoothed using a 4-mm FWHM Gaussian kernel. Signals from known nuisance variable (e.g., CSF, motion parameters) were not explicitly removed by regression. This information is used in the group independent component analysis (groupICA) to identify the independent components.

GroupICA was performed using an unconstrained mid-order, 28 independent components, model applied on the entirety of the dataset (43 young and 43 older healthy individuals) with the GIFT toolbox (GIFTv2.0, http://mialab.mrn.org/software/gift/), using the *Infomax* algorithm (Bell and Sejnowski, 1995), standard PCA type, and back-reconstruction using the GICA method. Reliability and consistency of the ICA algorithm were assessed using the ICASSO toolbox (http://www.cis.hut.fi/projects/ica/icasso/; Himberg et al., 2004), with 20 iterations using *RandInit* and *Bootstrap* methods. The usage of data-driven analysis approach allowed us to estimate functional connectivity without making any prior assumptions on how intrinsic activity is implemented.

### Network co-activation differences

Network co-activation differences between the two groups (young adults vs. older adults) were examined using a SPM two-sample *t*-test on the spatial distribution of the components (pDMN, aDMN, and vDMN). Statistical images (*t*-maps) were corrected for multiple-comparison using a cluster-wise correction. A minimum of 75 contiguous voxels was required for a cluster at *p* = 0.001 voxel-level to be considered significant, as computed by *3dClustSim* (AFNI, http://afni.nimh.nih.gov/afni/).

### Functional network connectivity (FNC)

Functional network connectivity analysis, an assessment of inter-network connectivity, reflective of between-network communication, were assessed for differences between the age groups (young and older adults) using the Functional Network Connectivity (FNC, FncVer2.3) toolbox, with maximal lag shift of 1 s. FNC was calculated following the procedure described by Jafri et al. (2008). Number of component-pairs (comp-pair) was obtained from a computation from the number of non-noise components (*n* = 19). In each population group, we identified high correlation component-pairs defined with a liberal threshold of Pearson's rho coefficient >0.3. Furthermore, only the overlap of high correlation component-pairs (*n* = 31) between the populations were statistically tested. Correction for multiple comparison was implemented using *FDR* approach following the procedure introduced by Storey (2002).

## Results

### DMN sub-components

**Figure 1:**
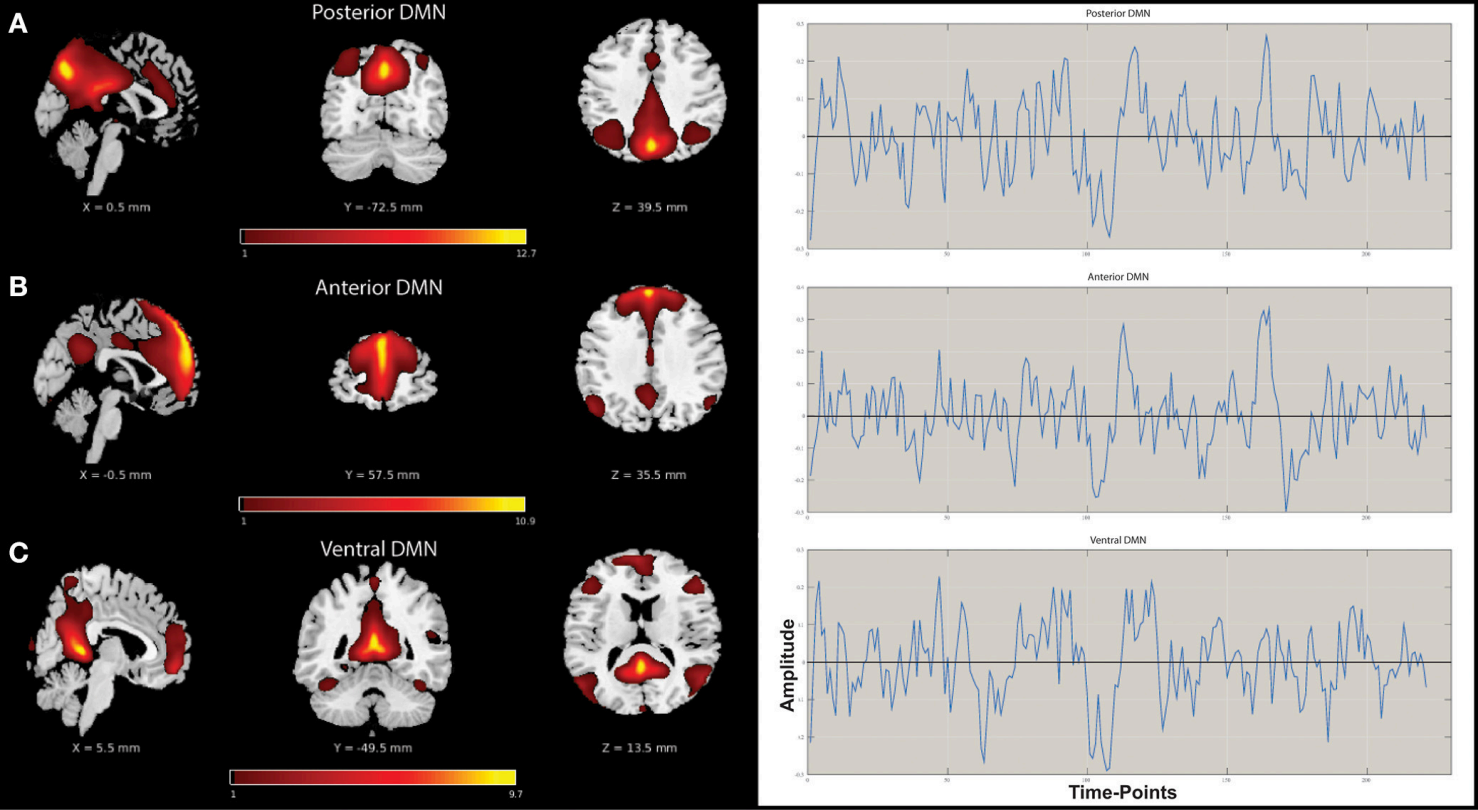
**Spatial distribution maps and associated time-courses of DMN subnetwork components**. **(A)** Posterior DMN (pDMN), **(B)** Anterior DMN (aDMN), and **(C)** Ventral DMN (vDMN).

A mid-order groupICA with 28 independent components explained 89.2% of the total observed variance and identified 19 non-noise components, including representations of the DMN, salience, attention, sensorimotor, and visual networks among others. Three components shared spatial distribution resembling that of the DMN and were tested for correlation and multiple-regression to a DMN-template (*dmn_mask_calhoun.nii;* Allen et al., 2011) with results presented in Table 2. The component most influenced by fluctuations of the precuneus/posterior cingulate cortex (pC/PCC) complex demonstrated the highest correlation (*r* = 0.524) with the template. The vDMN and aDMN components demonstrated comparatively lower correlations (*r* = 0.338 and *r* = 0.288, respectively) to the DMN-template. Robustness of each of the component were assessed by *(Power*_*LF*_*)/(Power*_*HF*_*)* ratio representing the strength of the resting-state signal (0.01–0.1 Hz range, “low-frequency”) to that of the observed higher frequency (0.1–0.192 Hz). Spatial distributions and time-courses of those DMN subnetwork components are presented in Figure 1.

**Table 2:** **Identification of DMN subnetwork components by correlation to the DMN spatial template**.

**Component**	**DMN-template correlation**	**Multiple regression**	***(Power _*LF*_)/ (Power _*HF*_)***
Posterior DMN (pDMN)	0.524	0.275	70.334
Ventral DMN (vDMN)	0.338	0.114	78.549
Anterior DMN (aDMN)	0.288	0.083	56.131
Salience network	0.233	0.054	90.126

After the three DMN components, the salience network component exhibited the next highest correlation to the DMN spatial template.

### Age-related DMN subnetwork co-activation differences

**Figure 2:**
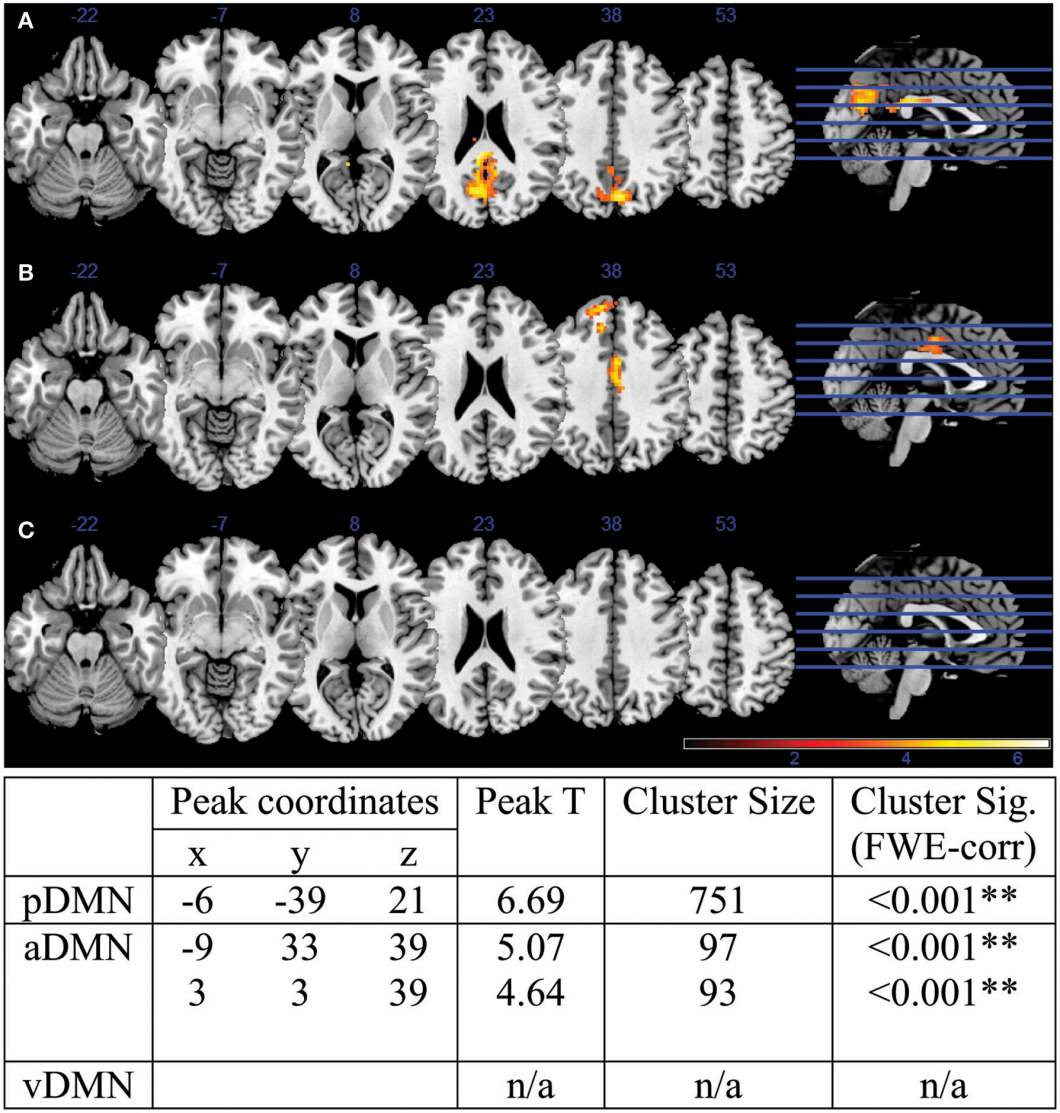
**DMN co-activation Young vs. Old contrast for (A) pDMN component, (B) aDMN component, and (C) vDMN component**. Attached table illustrates peak coordinates, peak *t*-statistics, as well as cluster statistics and cluster significance. Two of the three identified DMN components exhibited statistically different activation maps between the young and older adults: the pDMN and aDMN, with pDMN presenting 1 cluster of difference in the pC/PCC and aDMN presenting 2 cluster in the middle cingulum and mPFC over the whole brain.

A two-sample *t*-test revealed differences in co-activation maps in each of the three DMN subnetwork components. Corrected with *FWE*-correction, the older adult group exhibited lower network co-activation in the pDMN as well as in the aDMN components compared to the younger adults (Figure 2). One cluster in the pDMN component was particularly robust with a size of 751 voxels, with a peak *t*-statistic of 6.69 centered in the precuneus (MNI −6 –39 21). Differences were also observed in the aDMN component, with two smaller clusters of 97 voxels in the central cingulum, and another of 93 voxels in the medial PFC with peak *t*-statistic of 5.07 and 4.64, respectively. The group of older adults did not demonstrate any cluster of significance compared to the young adults in the vDMN component.

### Age-related inter-network connectivity differences

**Figure 3:**
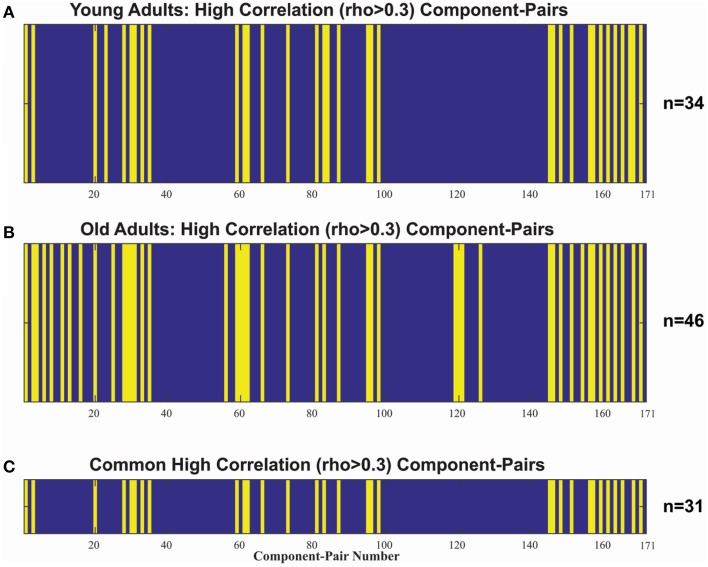
**Indexes of the 171 unique comp-pair transpose into a 1-dimension vector, detailing non-noise component-pairs with high correlations in (A) the young adult (18–30 y.o) group (*n* = 34), and (B) the older (>50 y.o.) adult group (*n* = 46)**. *Blue* denotes rho ≤ 0.3 and *Yellow* denotes rho > 0.3. **(C)** illustrates the 31 high-correlation component-pairs overlap between the two populations, which was used to compare the age-related inter-network correlation differences.

In addition to an assessment of within-network co-activation differences, we performed an assessment of between-component connectivity. For that purpose, we identified the component-pairs with high correlation (Pearson's rho > 0.3) for each of our populations (Figure 3). The older adult group showed a greater number of high correlation component-pairs, specifically 46 high-correlation component-pairs in the older adult group compared to only 34 in the younger group (Figures 3A,B). Thirty-one high-correlation component-pairs were common between the two age groups (Supplementary Material). Aside from those overlapping component-pairs across the two groups, three component-pairs reaching correlation threshold (Pearson's rho > 0.3) in the young adult group were not apparent in the older adul group, while 15 other component-pairs surged with high-correlations (Table 3).

**Table 3:** **High-correlation component-pairs gained and lost with aging**.

**Additional rho > 0.3 correlations in older Adults**						**Correlations that disappeared in older adults**	
**Number**	**Comp-Pair**	**Number**	**Comp-Pair**	**Number**	**Comp-Pair**	**Number**	**Comp-Pair**
4	V2-cereb	25	V1-amyg	121	Amyg-motor	23	V1-pDMN
6	V2-pDMN	29	V1-vAtt	126	Amyg-auditory	84	aDMN-vAtt
8	V2-amyg	56	vOcc-amyg	157	vAtt-salience	167	Salience-vAtt
11	V2-V3	60	vOcc-vlPFC(att)				
13	V2-motor	119	Amyg-V3				
16	V2-salience	120	Amyg-vlPFC(att)				

Purple–15 new component-pairs gained, Green—3 component-pairs lost, in the older adults.

**Figure 4:**
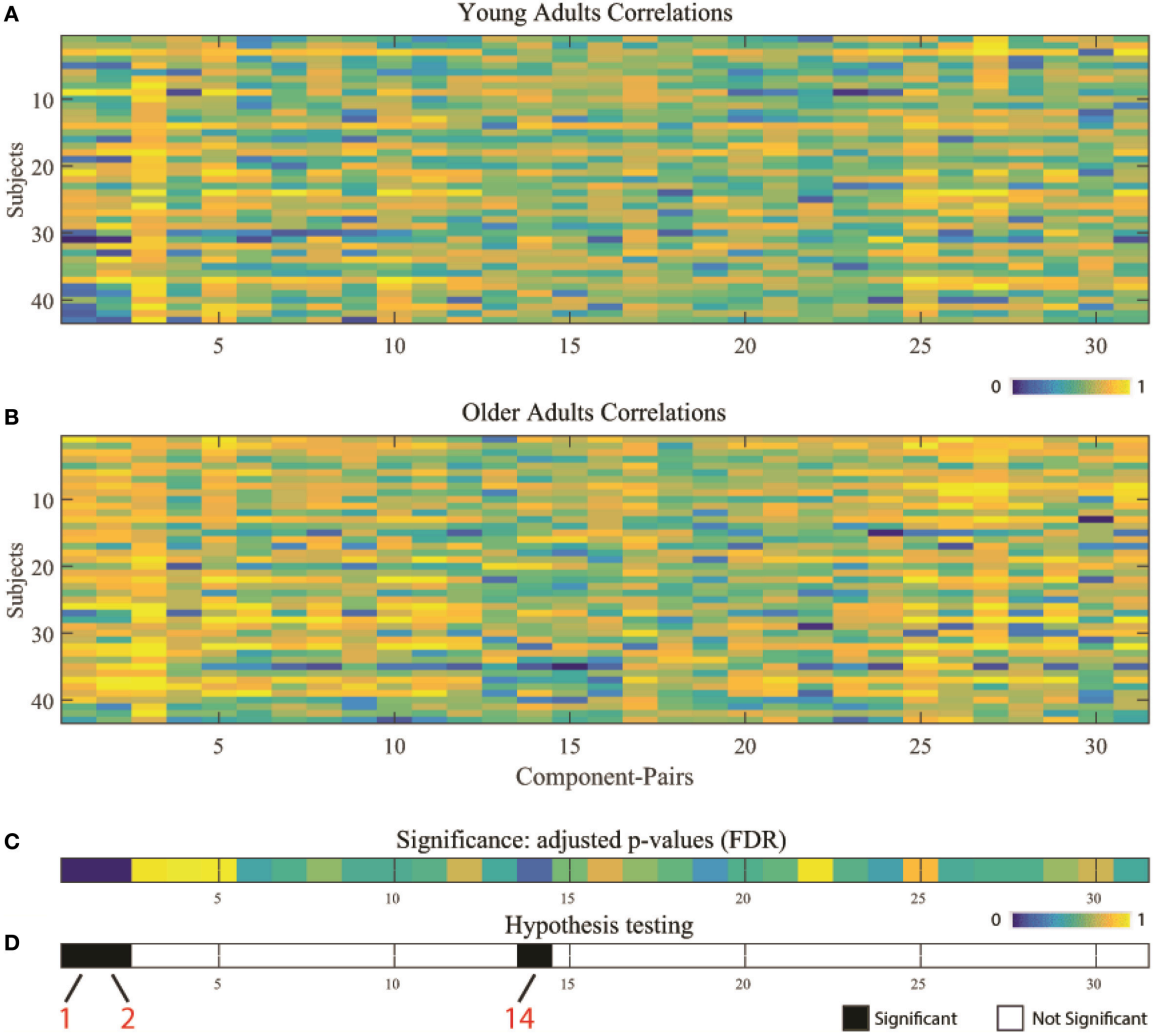
**Component-pairs correlation Pearson's rho values for individual subject by population group: (A) young adults, and (B) older adults**. **(C)** Illustrates results from two-sample *t*-tests between the two age groups across the 31 overlapping high-correlation component-pairs, corrected for multiple comparisons using *FDR* correction. **(D)** Presents the results of the hypothesis testing, with black illustrating significant differences (white = non-significant) between the two different age groups. Component-pairs 1 (V2–V1), 2 (V2–V4), and 14 (aDMN–pDMN) exhibited statistically significant differences. (*V1, visual area 1 component; V2, visual area 2 component; V4, visual area 4 component; aDMN, anterior DMN component; pDMN, posterior DMN component)*.

**Figure 5:**
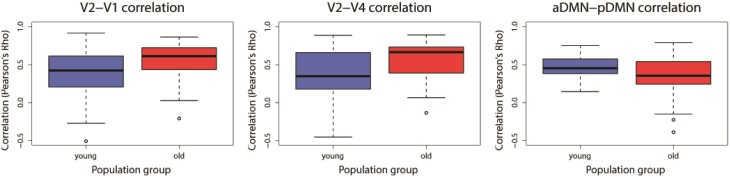
**Significant differences found for three component-pairs: [V2–V1], [V2–V4], and [aDMN–pDMN]**. *Blue* denotes the young adults and *Red* represents the older adults. Component-pairs V2–V1 and V2–V4 demonstrated an increase of the between-network correlation with age, while the aDMN–pDMN demonstrated a reduction of its between-network correlation. (*V1, visual area 1 component; V2, visual area 2 component; V4, visual area 4 component; aDMN, anterior DMN component; pDMN, posterior DMN component)*.

**Figure 6:**
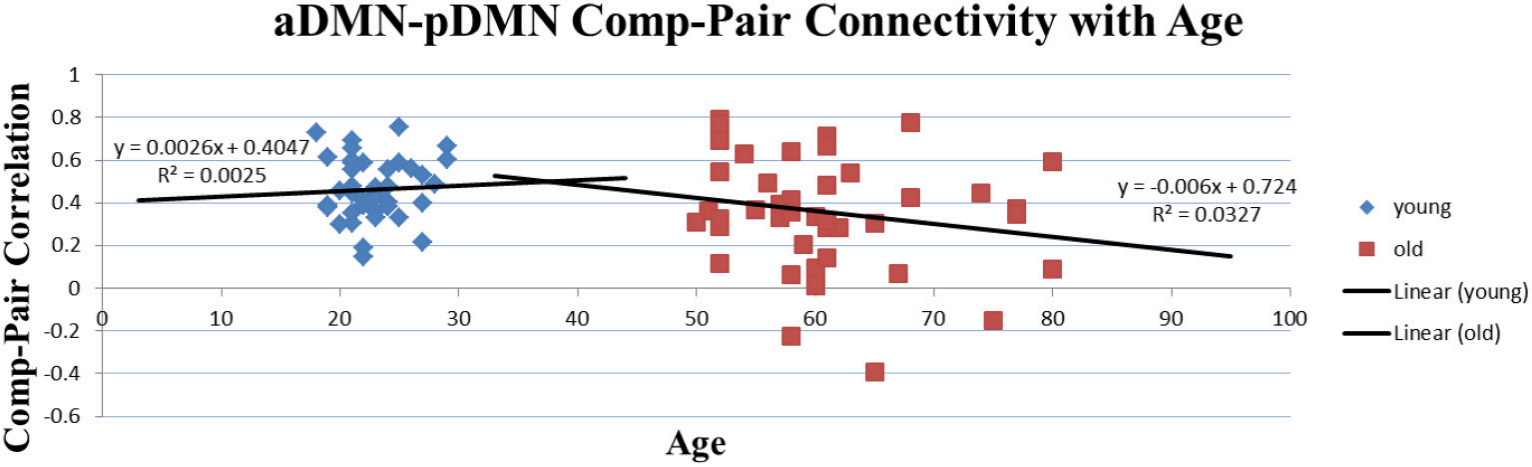
**Correlation of Age and Comp-pair Connectivity**. Though not significant and for illustrative purpose only, this plot presents a difference in the trajectory of the comp-pair connectivity with age, with an positive slope in the young adults and a negative slope in the older adults.

Two-sample *t*-tests were implemented for each of the 31 overlapping component-pairs. Three of those thirty-one component-pairs exhibited a significant difference between the two groups (corrected for multiple-comparison testing with *FDR*-correction; Table 4). These significant component-pairs were visual areas V2–V1 (“1”), visual areas V2–V4 (“2”), and aDMN-pDMN (“14”; Figure 4). Difference in component-pairs correlations are illustrated in Figure 5. The correlation coefficient in V2–V1 showed an age-related increase with mean rho of 0.358 in the young adult group to 0.550 in the older adult group (*p* < 0.01). The correlation coefficient in V2–V4 was also higher in the older adult group (0.352 in young adults vs. 0.567 in older adults, FDR-corrected *p* < 0.01). In contrast, the direction of the difference was opposite in the aDMN-pDMN correlation. Specifically, correlation between the aDMN and pDMN decreased from a mean rho of 0.464 in the young to 0.356 in the older adults, with *FDR*-corrected *p* < 0.05. Furthermore, a linear regression analysis of aDMN–pDMN comp-pair connectivity and age showed a pattern of a negative relationship between them, with young adults demonstrating a slight positive slope, while the older adults demonstrated a negative slope (Figure 6).

**Table 4:** **Component-Pair Correlations and *FDR*-adjusted *p*-values for the three component-pairs exhibiting statistical difference between the two population groups**.

**Comp-pair Number**	**Component-pair**	**Corr. value in young**	**Corr. value in older**	***p*-Values**
1	V2–V1	0.358	0.550	*p* < 0.01^**^
2	V2–V4	0.352	0.567	*p* < 0.01^**^
14	aDMN–pDMN	0.464	0.356	*p* < 0.05^*^

V1, visual area 1 component; V2, visual area 2 component; V4, visual area 4 component; aDMN, anterior DMN component; pDMN, posterior DMN component.

* significant,

** highly significant.

## Discussion

Brain aging involves an evolution of cortical architecture, an alteration of the functional framework to adapt to the environment likely including compensatory mechanisms such as reduction of functional segregation, and a modification of the scaffold in response to structural deficits. This study characterized the effects of aging on intrinsic brain activity in the DMN and on inter-network communication (between-network connectivity) within the cortical system. Through the use of component analysis, we have presented an investigation of the subnetworks of the DMN, finding that: (1) of the three isolated subnetworks of the DMN, two (pDMN and aDMN) exhibited significant differences in network co-activation between the two population groups, and (2) the interaction between those two subnetworks may also become interrupted with age.

The default-mode network has consistently shown disrupted functional connectivity in aging and clinical populations (Andrews-Hanna et al., 2007; Damoiseaux et al., 2008; Greicius, 2008; Broyd et al., 2009). Using a component-based approach, our data, similarly, demonstrated reduced functional connectivity within the DMN. Two of the three identified subnetworks were found to exhibit reduced within-component network co-activation. In the pDMN, reduction of co-activation occurred most strongly in the pC/PCC complex, a known pivotal core and strong oscillator within the DMN (Fransson and Marrelec, 2008). This region is also one of the most metabolically active areas in a healthy brain, and is vulnerable to disruption in older and clinical populations (Raichle et al., 2001). Moreover, reduction of activity in this area has been previously associated with cognitive decline in advanced aging and various clinical populations such as Alzheimer's dementia (Lustig et al., 2003; Grady et al., 2006; Persson et al., 2007). Similarly, in the aDMN subsystem, a reduction in network co-activation was also found in concordance with other previous reports (Rombouts et al., 2005; Damoiseaux et al., 2008). It has been previously suggested that the DMN can be divided into two primary subsystems (Buckner et al., 2008): a posterior subsystem including the pC/PCC, bilateral IPL and the MTL for episodic memory retrieval (Wagner et al., 2005), and an anterior subsystem with the anterior regions of the DMN (the dorsal and ventral mPFC), and including the pC/PCC complex, involved in self-referential mental thoughts (Gusnard et al., 2001b; Schmitz et al., 2004; Johnson et al., 2005). In this model, the pC/PCC has been suggested to act as a convergence node where information processing in the two subsystems is integrated (Fransson and Marrelec, 2008). It is possible that with aging, these two subsystems of the DMN become disrupted in their pattern of activation, resulting in cognitive, and behavioral deficits associated with increasing age.

Additionally, in this study we presented an investigation of the interaction of brain network components using a between-network connectivity analysis which assessed the correlations between component pairs. Out of the 171 component-pairs, the older adults demonstrated more numerous high-correlation pairs (Pearson's *rho* < 0.3) compared to younger adults. In the older group, 46 component-pairs exceeding rho = 0.3 were recorded, while the younger group only exhibited 34 of those pairs. The higher number suggests that greater between-network communication is occurring in the older adults. This is in accord with the reduction of functional specialization, or “dedifferentiation” model of aging (Baltes and Lindenberger, 1997; Li et al., 2001; Park et al., 2004), where functional units exhibit reduced functional specificity. Our results also supports the reduction of functional segregation in a graph-theoretic framework (Meunier et al., 2009, 2014; Chan et al., 2014), an extension of the “dedifferentiation” model, where within-network connectivity is reduced in return for an increase of connectivity between networks, and where modularity of the cortical system is reduced with aging. Moreover, our finding may also reflect the phenomena of “hyper-binding” in older adults, where older adults are both more likely to encode and less likely to suppress irrelevant distracter information in situations when relevant information must be retrieved and brought to attention (Campbell et al., 2010, 2014; Biss et al., 2013; Lustig and Jantz, 2015). In other words, older adults are both more likely to encode and less likely to suppress distracter information of no value. At the level of the network, binding occurs with synchronization of oscillations between regions. The existence of a hyper-binding state would suggest the presence of a supporting hyper-connected system, represented here by more numerous between-network connections.

Numerous comp-pair correlation differences were recorded between the two groups. In the older population, high-correlation comp-pairs (*n* = 15) were gained, while some other comp-pairs (*n* = 3) were lost (Table 3). However, for our investigation, we focused on the high-correlation comp-pairs present in both populations, verifying a measure of stability within the component-pair. Reducing the assessment to the 31 overlapping high-correlation component-pairs, we found 3 component-pairs that were significantly different across the two population groups: V2–V1, V2–V4, and aDMN-pDMN. Correlations between visual areas V2–V1 and V2–V4 were higher in the older adults and may represent a higher integration of V2 with the other visual subnetworks, in line with previous reports of reduced neural specialization of the visual system with aging (Park et al., 2004, 2012). Park et al. (2012) have suggested a broadening of the tuning curves of the specific visual neurons as a possible mechanism underlying the dedifferentiation process in the ventral visual system. Here, we have demonstrated that the component-pairs of the visual systems are more correlated with older age, facilitating communication and functional overlap between the visual subsystems.

Correlation between the components of aDMN and pDMN was found to be reduced in the older adult group. Previously, network co-activation reduction within each of those components (aDMN and pDMN; Damoiseaux et al., 2008) and reduction in pair-wise connectivity between an anterior (mPFC) and a posterior (PCC) region of the DMN (Andrews-Hanna et al., 2007; Buckner et al., 2008) in older subjects have been postulated independently. Here, we showed via component analysis that in addition to the reduction of network co-activation within the subnetworks, the interaction between networks is reduced as well. Independent component analysis is a data-driven, model-free approach, which removes the susceptibility involved in the placing of seed regions for pair-wise correlation analyses). To our knowledge, this is the first study to show this reduction in correlation between these two DMN subsystems by a component analysis approach, and demonstrates that this inter-network connectivity between the anterior and posterior subsystems of the DMN is among the most disrupted in the aging cortical system.

Another advantage of ICA compared to seed-based approaches is that it is less vulnerable to motion-induced signal. Specifically, head motion has been shown to greatly influence functional connectivity of the rs-fMRI signals (Van Dijk et al., 2012), with spatial blurring from motion noise suggested to increase local correlation of the signal while decreasing the strength of long-range coupling to anatomically-specific regions. Opportunely, ICA presents less susceptibility to artefactual effects from noise, as the ICA provides the ability to identify and remove non-RSN components from the rs-fMRI signal (Birn et al., 2008; Murphy et al., 2009; Cole et al., 2010). Moreover, the spatial overlap of the aDMN and pDMN components further reduces the contribution of motion on each independent DMN component.

Our understanding of the causes and consequences of the decreased antero-posterior DMN connectivity remains limited despite the unequivocal evidence of a vulnerability of DMN connectivity in aging. Tomasi and Volkow (2012) did also demonstrate a pronounced long-range functional connectivity density (FCD) decrease within the DMN, associated with aging. This finding is, however, not in accord with the reduction of network segregation hypothesis (Meunier et al., 2009; Chan et al., 2014), where an increase in between-network correlation is predicted. Instead, Vidal-Piñeiro et al. (2014) have suggested that decreases in this mPFC-PCC connectivity may not be associated with compensatory mechanisms; but rather reflect aging of the brain architecture, ultimately contributing to a decline in cognitive function. Such a reduction of inter-network correlations between the aDMN and pDMN subnetworks may then be due to changes in the structural architecture underlying functional connectivity. It is increasingly accepted that anatomical connectivity supports functional connectivity in the resting state (Skudlarski et al., 2008; Honey et al., 2009; Horn et al., 2014). In general, older age has been associated with white matter changes in the corona radiata, superior longitudinal fasciculus, and cingulum (Pfefferbaum et al., 2000, 2005; Head et al., 2004). Evidence suggests the functional connectivity between posterior midline structures and the medial prefrontal cortex are related to white matter microstructure (Greicius et al., 2009; Vidal-Piñeiro et al., 2014), and age-associated compromise of the white matter microstructure were in turn associated with altered anterio-posterior connectivity (Andrews-Hanna et al., 2007). Therefore, it is possible that some of the observed changes in our study may have been due to alterations in structural connectivity; future studies incorporating white matter imaging measures will be needed to address this.

### Limitations

A few limitations deserve mention. First, despite the advantage of ICA in addressing the issue of motion in resting-state fMRI, influence of motion in resting-state connectivity analysis remains an issue, especially with regards to long-distance connections. In the investigation of connectivity difference in aging, influence of motion is a particular issue, as high levels of head motion could drive a reduction of this anterio-posterior connectivity (Van Dijk et al., 2012). However, with an analysis of the correlation between the components of the aDMN and pDMN, both sharing a large spatial overlap, encompassing regions of the DMN, the influence of motion is further reduced, though not totally removed.

Another limitation concerns the possible confound of cardiac and respiratory pulsations. Aliasing of those physiological signals into the resting-state frequencies is known to occur, which can contribute to the synchronization of the intrinsic low-frequency oscillations (Birn et al., 2006; Biswal et al., 2007). However, it has been shown that by applying multiple regression techniques like ICA, cardiac and respiratory induced signal variations can be separated from signal fluctuations of interest (Beckmann et al., 2005; De Luca et al., 2006; Fukunaga et al., 2006; Birn et al., 2008), thus somewhat reducing this concern. Finally, it is likely that incorporating additional modalities such as diffusion tensor imaging (DTI) could shed light on the extent to which loss of structural connectivity underlies changes in age-associated functional connectivity, particularly the loss of synchronization between aDMN and pDMN observed in this study.

## Conclusions

In summary, we demonstrated through a data-driven approach that not only is intrinsic activity of the DMN affected by aging, but the interactions between the posterior and anterior components of the DMN are affected as well. In line with previous studies, the pDMN and aDMN demonstrated a reduction in network co-activation in the older adult group. We also showed a more numerous count of high-correlation component-pairs, accompanied by a higher correlation within the networks of the visual system. This finding may indicate a more integrated/less segregated system in older subjects. Moreover, we found a reduction in aDMN–pDMN component connectivity in the older adult group, demonstrating a disconnection of these two DMN subsystems with age. However, follow-up studies are needed to determine the mechanism by which this occurs.

## Authors contributions

CL is the corresponding author for the manuscript and have participated in the data collection, data analysis, and writing. PM and VN assisted in the development of the analysis approach as well as writing. BB and RB provided their expertise in the field of brain aging and resting-state fMRI, respectively, and assisted with the writing. MM and VP are the two lead PI of this investigation.

### Conflict of interest statement

The authors declare that the research was conducted in the absence of any commercial or financial relationships that could be construed as a potential conflict of interest.
