# Supplementary material for: Age-Related Changes in Inter-Network Connectivity by Component Analysis
Source: Front Aging Neurosci. 2015 Dec 24;7:237. doi: 10.3389/fnagi.2015.00237 (PMC4689781; doi:10.3389/fnagi.2015.00237)
Supplement: Supplementary file 1 [file Image1.pdf]

Supplemental Material A

#	Comp-Pair	#	Comp-Pair	#	Comp-Pair	#	Comp-Pair	#	Comp-Pair	#	Comp-Pair
**1**	V2-V1	**33**	V1-salience	**73**	Cereb-V3	**98**	pDMN-V3	**157**	Motor-vDMN	**170**	Salience-auditory
**3**	V2-V4	**35**	V1-auditory	**81**	aDMN-pDMN	**145**	V3-motor	**159**	motor-salience		
**20**	V1-V4	**59**	vOcc-V3	**83**	aDMN-att	**146**	V3-vDMN	**161**	motor-auditory		
**28**	V1-V3	**61**	vOcc-motor	**87**	aDMN-motor	**148**	V3-salience	**163**	vDMN-salience		
**30**	V1-V5/MT	**62**	vOcc-vDMN	**95**	pDMN-vDMN	**151**	V5/MT-motor	**165**	vDMN-auditory		
**31**	V1-motor	**66**	vOcc-auditory	**96**	pDMN-att	**159**	V5/MT-auditory	**168**	salience-auditory		

Supplemental A. Thirty-one overlapping high-correlation pairs (rho>0.3). Shaded in red are comp-pairs where older adults showed significant difference in the strength of the correlation compared to the younger adult group.
